# Transmembrane helices 5 and 12 control transport dynamics, substrate affinity, and specificity in the elevator-type UapA transporter

**DOI:** 10.1093/genetics/iyac107

**Published:** 2022-07-27

**Authors:** Dimitris Dimakis, Yiannis Pyrris, George Diallinas

**Affiliations:** Department of Biology, National and Kapodistrian University of Athens, 15784 Athens, Greece; Department of Biology, National and Kapodistrian University of Athens, 15784 Athens, Greece; Department of Biology, National and Kapodistrian University of Athens, 15784 Athens, Greece; Institute of Molecular Biology and Biotechnology, Foundation for Research and Technology, 70013 Heraklion, Greece

**Keywords:** *Aspergillus nidulans*, purine, uric acid, NAT/NCS2, structure-function

## Abstract

An increasing number of solute transporters have been shown to function with the so-called *sliding-elevator* mechanism. Despite structural and functional differences, all elevator-type transporters use a common mechanism of substrate translocation via reversible movements of a mobile core domain (the *elevator*) hosting the substrate binding site along a rigid scaffold domain stably anchored in the plasma membrane via homodimerization. One of the best-studied elevator transporters is the UapA uric acid-xanthine/H^+^ symporter of the filamentous fungus *Aspergillus nidulans*. Here, we present a genetic analysis for deciphering the role of transmembrane segments (TMS) 5 and 12 in UapA transport function. We show that specific residues in both TMS5 and TMS12 control, negatively or positively, the dynamics of transport, but also substrate binding affinity and specificity. More specifically, mutations in TMS5 can lead not only to increased rate of transport but also to an inactive transporter due to high-affinity substrate-trapping, whereas mutations in TMS12 lead to apparently uncontrolled sliding and broadened specificity, leading in specific cases to UapA-mediated purine toxicity. Our findings shed new light on how elevator transporters function and how this knowledge can be applied to genetically modify their transport characteristics.

## Introduction

Transporters are transmembrane proteins that mediate the selective translocation of nutrients, metabolites, or drugs across cellular membranes. Their activity is essential for cell survival, division, and differentiation and consequently for the life of all organisms. Thus transporter malfunction is associated with several diseases, such as cystic fibrosis, diabetes, or neurodegeneration. Transporters, despite their structural and functional differences, alternate in structurally distinct conformations during transport: outward-facing open (substrate reception), outward-facing occluded (substrate oriented in the major binding site and closure of outer gate), fully occluded (substrate stabilized in a major binding site), inward-facing occluded (substrate binding induces inward conformation), and inward-facing open (inner gate opens and substrate released; Penmatsa and Gouaux 2014; Simmons *et al.* 2014; Coincon *et al.* 2016; Drew and Boudker 2016). Based on their structure, mechanism of function, and evolutionary origin, most transporters fall into 3 distinct groups, known as the Major Facilitator Superfamily, the Amino Acid-Polyamine-Organocation superfamily, and a more structurally diverse group of transporters operating by the so-called sliding-elevator mechanism of transport, which necessitates functional dimerization (Drew and Boudker 2016).

Elevator-type transporters (symporters and antiporters or exchangers) possess a motile substrate binding site included in a core or elevator domain that moves through the membrane as a rigid body, translocating the substrate(s) from one side of the membrane to the other. The movement of the core/elevator domain takes place along a relatively rigid scaffold domain, anchored in the plasma membrane (PM) via homodimerization and specific interactions with lipids (Lee *et al.* 2013; Alguel *et al.* 2016; Byrne 2017; Pyle *et al.* 2018; Garaeva and Slotboom 2020; Diallinas 2021). The basic aspects of this mechanism of transport have been deciphered by comparing outward- and inward-facing cryo-electron microscopy (EM) and X-ray crystallography structures of several elevator-type transporters (Lee *et al.* 2013; Geertsma *et al.* 2015; Alguel *et al.* 2016; Coincon *et al.* 2016; Thurtle-Schmidt and Stroud 2016; Ficici *et al.* 2017; Yu *et al.* 2017). Structural studies on elevator-type transporters have been complemented and supported by extensive mutational and functional in vivo studies in UapA, a uric acid/xanthine transporter of the filamentous fungus *Aspergillus nidulans* (Koukaki *et al.* 2005; Pantazopoulou and Diallinas 2006; Vlanti *et al.* 2006; Papageorgiou *et al.* 2008; Kosti *et al.* 2010; Amillis *et al.* 2011; Kourkoulou *et al.* 2019, 2021). In particular, the genetic studies in UapA have contributed in understanding how substrate specificity is determined, an issue hard to approach solely via structural studies. Most interestingly, specificity mutations that broaden the ability of UapA to bind all purines and uracil do not modify residues of the substrate binding site [i.e. in transmembrane segments TMS3, TMS8, or TMS10], as might have been expected. Instead, they concern residues in TMS12, TMS13, or TMS14, which are helices of the rather rigid scaffold/dimerization domain. Other specificity mutations affected residues in flexible loops linking the core/elevator and the scaffold domains. On the other hand, mutations in residues involved in direct substrate coordination and transport, located in TMS3, TMS8, and TMS10 of the movable elevator, alter the transport kinetics of UapA in respect to its native substrates, but generally do not modify specificity for nucleobases other than physiological substrates [for a recent review see Diallinas (2016)].

Homology modeling of the outward-facing conformation and comparison with inward-facing crystal structure of UapA provided an explanation on how specificity can be modified by affecting the dynamics of sliding of the core/elevator domain (Diallinas 2021). In the outward-facing conformation and in the absence of substrate the core/elevator domain seems to be “locked” by tight interactions with specific residues in the scaffold domain and in particular with TMS12 and TMS14. Protonation of the substrate binding site and subsequent substrate binding seems to ‘unlock’ the sliding of the elevator by altering the polar character of the substrate binding site, which can now slide toward the cytoplasmic side of the PM along the interface with the scaffold domain, and in particular along TMS12 and TMS5. Through this model, specificity mutations can be rationalized as amino acid modifications that somehow facilitate sliding or uncouple it from the requirement of high-affinity substrate binding. As a consequence, any purine or other structurally similar solute that has access and fits into the binding site cavity, even with low affinity, can be transported by UapA.

In this work, we investigate the mechanism of UapA transport function by designing and functionally analyzing novel mutations in TMS5 and TMS12, which form the sliding interface of the elevator. We present evidence that specific residues in both TMS5 and TMS12 control negatively or positively the dynamics of sliding of the elevator and thus also modify transport kinetics and specificity of UapA.

## Materials and methods

### Media, strains, and growth conditions

Standard complete (CM) and minimal media (MM) for *A. nidulans* growth were used. Media and supplemented auxotrophies were used at the concentrations given in http://www.fgsc.net (McCluskey *et al.* 2010). Glucose 1% (w/v) was used as carbon source. Ten millimolar of sodium nitrate (NO_3_) was used as a standard nitrogen source. Nucleobases and toxic analogs were used at the following final concentrations: 5-FU, 8-azaguanine, and oxypurinol at 100 μΜ; uric acid, xanthine, adenine, hypoxanthine, and guanine at 0.5–2.0 mM. All media and chemical reagents were obtained from Sigma-Aldrich (Life Science Chemilab SA, Hellas) or AppliChem (Bioline Scientific SA, Hellas). A *ΔfurD::riboB ΔfurA::riboB ΔfcyB::argB ΔazgA ΔuapA ΔuapC::AfpyrG ΔcntA::riboB pabaA1 pantoB100* mutant strain, named Δ7, was the recipient strain in transformations with plasmids carrying *uapA* alleles based on complementation of the pantothenic acid auxotrophy *pantoB100* (Krypotou and Diallinas 2014). *pabaA1* is a paraminobenzoic acid auxotrophy. *A. nidulans* protoplast isolation and transformation were performed as previously described (Koukaki *et al.* 2003). Growth tests were performed at 25°C for 96 h, at pH 6.8.

### Standard molecular biology manipulations and plasmid construction

Genomic DNA extraction from *A. nidulans* was performed as described in FGSC (http://www.fgsc.net). Plasmids, prepared in *Escherichia coli*, and DNA restriction or PCR fragments were purified from agarose 1% gels with the Nucleospin Plasmid Kit or Nucleospin ExtractII kit, according to the manufacturer’s instructions (Macherey-Nagel, Lab Supplies Scientific SA, Hellas). Standard PCR reactions were performed using KAPATaq DNA polymerase (Kapa Biosystems). PCR products used for cloning, sequencing, and reintroduction by transformation in *A. nidulans* were amplified by a high fidelity KAPA HiFi HotStart Ready Mix (Kapa Biosystems) polymerase. DNA sequences were determined by VBC-Genomics (Vienna, Austria). Site-directed mutagenesis was carried out according to the instructions accompanying the Quik-Change Site-Directed Mutagenesis Kit (Agilent Technologies, Stratagene). The principal vector used for most *A. nidulans* mutants is a modified pGEM-T-easy vector carrying a version of the *gpdA* promoter, the *trpC* 3′ termination region, and the *panB* selection marker (Krypotou *et al.* 2015). Mutations were constructed by oligonucleotide-directed mutagenesis or appropriate forward and reverse primers (Supplementary Table 1).

### Protein model Construction

The inward-facing UapA model PDB ID is 5i6c. The outward-facing model of UapA was generated using as template the Band3 anion exchanger structural homolog crystallized in the outward conformation, as described in Kourkoulou *et al.* (2021). The models shown here are presented with PyMOL 2.5 (https://pymol.org).

### Transport assays

Kinetic analysis of wt and mutant UapA was measured by estimating uptake rates of [^3^H]-xanthine uptake (40 Ci mmol^−1^, Moravek Biochemicals, CA, USA), as previously described in Krypotou and Diallinas (2014). In brief, [^3^H]-xanthine uptake was assayed in *A. nidulans* conidiospores germinating for 4 h at 37°C, at 140 rpm, in liquid MM, pH 6.8. Initial velocities were measured on 10^7^ conidiospores/100 μL by incubation with concentration of 0.3 μΜ of [^3^H]-xanthine at 37°C. For the competition experiments, initial uptake rates of [^3^H]-xanthine were measured in the simultaneous presence increasing concentrations (1 μΜ to 1 mM) of various putative nucleobase-related inhibitors. All transport assays were carried out at least in 2 independent experiments and the measurements in triplicate. Standard deviation was <20%. Results were analyzed in GraphPad Prism software. The way the uptake kinetics assays are performed is described in Krypotou and Diallinas (2014). In brief, depending on the lot of radiolabeled xanthine used (usually at 0.2–2.0 μM xanthine), the stock solution is either diluted (for concentrations lower than the stock) or is mixed with increasing concentration of nonlabeled xanthine (for concentrations higher of 0.3 μM) to achieve serial concentrations up to 1 or 2 mM. Thus, in all cases, at least 3 concentration points below and 4–5 over the established *IC*_50_/*K*_m_/*K*_i_ are included.

### Epifluorescence microscopy

Samples for standard epifluorescence microscopy were prepared as previously described (Gournas *et al.* 2010). In brief, sterile 35 mm l-dishes with a glass bottom (Ibidi, Germany) containing liquid MM supplemented with NaNO_3_ and 1% glucose were inoculated from a spore solution and incubated for 16 h at 25°C. The images were obtained using an inverted Zeiss Axio Observer Z1 equipped with an Axio Cam HR R3 camera. Image processing and contrast adjustment were made using the ZEN 2012 software while further processing of the TIFF files was made using Adobe Photoshop CS3 software for brightness adjustment, rotation, alignment, and annotation.

## Results and discussion

### TMS5 mutations

**Fig. 1.**
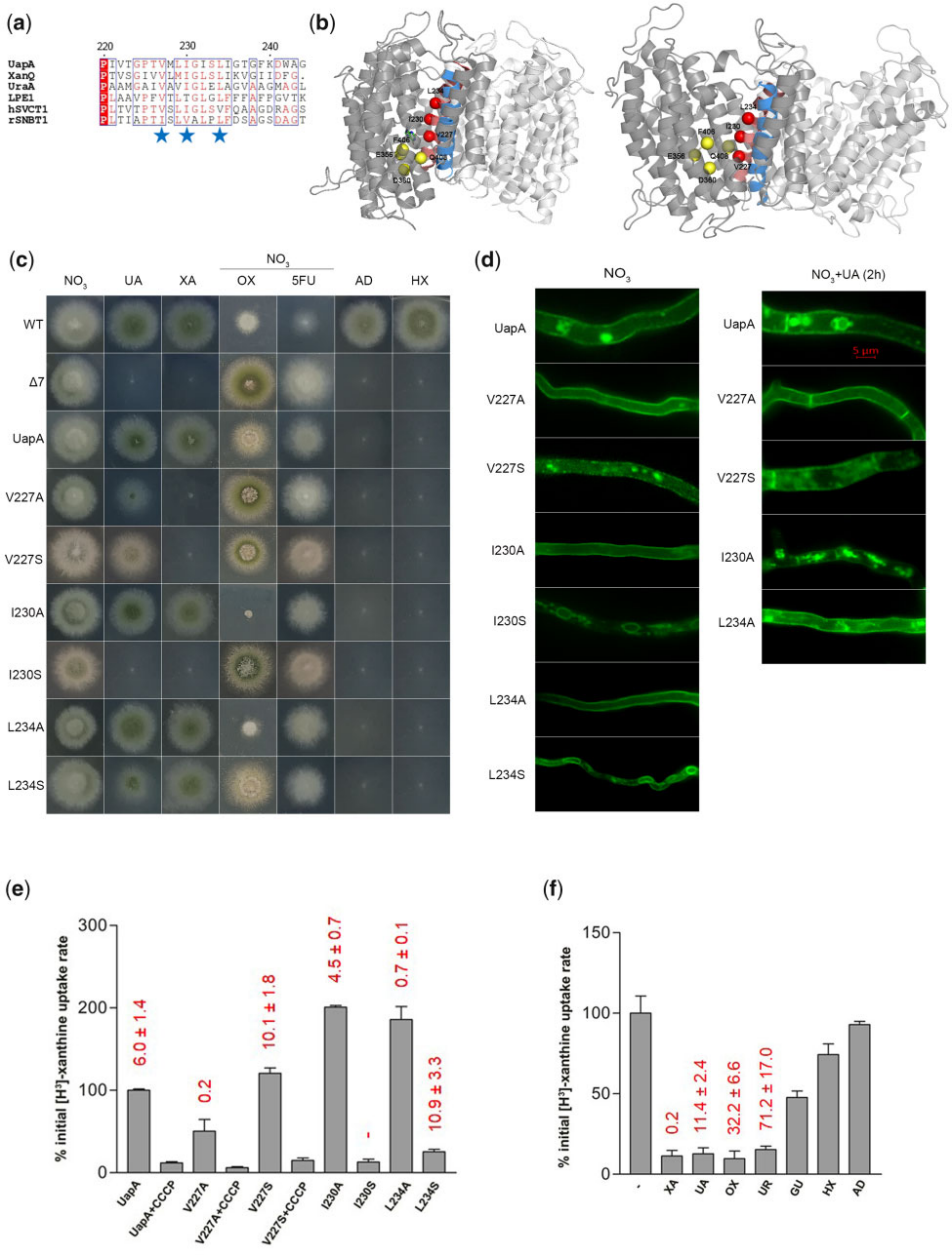
Functional analysis of TMS5 mutants. a) TMS5 sequence alignment of UapA, XanQ, UraA, LPE1, hSVCT1, and rSNBT1. XanQ and UraA are xanthine and uracil transporters, respectively, in *Escherichia coli*. LPE1 is a maize xanthine-uric acid transporter. hSVCT1 is a human L-ascorbate transporter. rSNBT1 is a rat purine-uracil transporter (Gournas *et al.* 2008). Blue stars indicate mutated amino acids. b) Topological models of inward-facing (left) and outward-facing (right) conformations of the UapA dimer highlighting TMS5 (red) and TMS12 (blue) at the interface with the core/elevator domain. Red spheres indicate the positions of the residues mutated (V227, I230, and L234). Yellow spheres represent residues in the core/elevator domain, directly involved in substrate binding (E356 and D360 in TMS8 and F406 and Q408 in TMS10). For clarity, annotations are shown in one of the 2 UapA monomers (deeper grey). Notice the downwards sliding of the core/elevator domain in the inward-facing conformer. c) Growth tests of UapA mutants on MM with purines as N source or nucleobase toxic analogs. UA, uric acid; XA, xanthine; 5FU, 5-flouoruracil; AD, adenine; HX, hypoxanthine; OX, oxypurinol. Growth on 10 mM sodium nitrate (${\text{NO}}_{3}^{-}$) is included as a standard N source control. Purine concentration as N sources is 0.5 mM. Concentration of toxic analogs is 100 μΜ. All tests are performed at 25°C and pH 6.8. Three control strains are included in the growth tests. WT is a wt strain possessing all relative endogenous nucleobase-related transporters. Δ7 is a strain lacking all 7 major nucleobase transporters (Krypotou and Diallinas 2014). UapA is a Δ7 strain expressing wt UapA functionally tagged with GFP. All UapA mutant versions shown are expressed and analyzed in the Δ7 strain. d) Epifluorescence microscopy showing the subcellular localization of UapA mutants and wt UapA in the presence of ${\text{NO}}_{3}^{-}$ as N source (left). Notice the perinuclear localization (rings) of I230S and L234S indicative of ER retention, and the increased endosomal localization of V227S linked to enhanced endocytic turnover. A similar experiment with wt UapA, V227A, V227S, I230A, and L234A in the presence of substrate (1 mM UA for 2 h) is shown in the right panel. Notice the PM stability of V227A relative to the wt UapA and the other mutants. e) Relative ^3^H-xanthine (0.3 μΜ) transport rates of all mutants analyzed expressed as percentages of initial uptake rates (V) compared to the wt UapA (UapA) rate. The time point used for the initial uptake rate is 1 min. CCCP is a proton gradient uncoupler inhibiting wt UapA activity. UapA initial uptake rate is arbitrarily taken as 100%. *K_m_* values (μΜ) for xanthine, estimated as described in Krypotou and Diallinas (2014), are shown at the top of histograms. Results are averages of 3 measurements for each concentration point. SD was less than 20%. f) Relative ^3^H-xanthine (0.3 μΜ) transport rates of strain V227A in the absence or presence of excess (2 mM) unlabeled nucleobases (UR is uracil and GU is guanine), expressed as percentages of initial uptake rates (*V*) compared to the rate of V227A in the absence of unlabeled nucleobases, considered as 100%. *K_m_* and *K_i_* values (μΜ) for XA, UA, OX, and UR are shown at the top of the histograms and were measured as described in Krypotou and Diallinas (2014). Results are averages of 3 measurements for each concentration point. SD was less than 20%.

The crystal structure and modeling of UapA have shown that TMS5 and TMS12 form the interface along which the core/elevator domain slides and mediates substrate/H^+^ symport. Specificity mutations have been isolated via unbiased genetic screens along TMS12 (Papageorgiou *et al.* 2008; Kosti *et al.* 2010), which is in line with the proposed model on how the dynamics of sliding might affect the selectivity of UapA, enabling non-native substrates to be recognized (Diallinas 2021). Curiously, no specificity mutation has been isolated in TMS5. This seems rather unexpected considering also that TMS5 is a relatively well-conserved TMS in UapA, especially in respect to large aliphatic residues (Val, Ile, Leu; Fig. 1a). The single known mutation in TMS5 is L234M, which has been isolated as a genetic suppressor that stabilizes the structure of UapA in a dimerization defective mutant (Kourkoulou *et al.* 2019). The L234M mutant has a wt-like affinity for native substrates, moderately reduced transport rate and leads to no substrate specificity modification.

To explore whether residues in TMS5 might affect the dynamics and/or specificity of transport, we constructed and functionally analyzed the following new mutants: V227A, V227S, I230A, I230S, L234A, and L234S. The residues modified are located at the interface of the TMS5 helix overlooking the substrate binding site in the sliding-elevator domain, and are all extremely conserved in the Nucleobase Ascorbate Transporter (NAT) family (Diallinas and Gournas 2008; Gournas *et al.* 2008), where UapA belongs (Fig. 1b). The mutated UapA versions were introduced and analyzed in a genetic background lacking detectable nucleobase-related transport activities due to null mutations in all relevant transporter genes (Δ7), as described in Kourkoulou *et al.* (2021). *A. nidulans* can grow on purines as N sources and is sensitive to several toxic analogs of nucleobases allowing direct assessment of UapA mutant versions expressed in Δ7 (Fig. 1c). At the physiological temperature of *A. nidulans*, that is 25°C, mutants I230A and L234A showed normal growth on media with uric acid and xanthine, similarly to an isogenic control strain expressing wt UapA, while L234S showed moderately reduced growth on these purines, and especially on uric acid. I230S scored as a loss-of-function mutation, similar to a strain lacking UapA. Both mutations in V227 led to inability for growth on xanthine and to significantly reduced growth on uric acid, suggesting they are partial loss-of-function mutations, affecting mostly xanthine recognition and/or transport. We also examined whether TMS5 mutations affected UapA-mediated sensitivity to oxypurinol, a toxic purine analog known to be specifically recognized by UapA. I230A and L234A showed increased sensitivity to oxypurinol compared to the wt UapA control, suggesting enhanced activity or/and increased presence of UapA in the PM. In contrast, V227A and Ser-substituted mutants showed reduced sensitivity to oxypurinol. Finally, all mutants tested were resistant to 5-FU (toxic analog of uracil) and could not grow on adenine or hypoxanthine, similar to wt UapA (Fig. 1c). A similar growth phenotype was found when the growth tests were performed at 37°C, except mutant V227S, which exhibited the capacity to grow on uric acid and xanthine at 37°C, suggesting that this is a cryosensitive mutation (see Supplementary Fig. 1).

To test whether the growth phenotypes are related to UapA biogenesis and PM localization, we performed epifluorescence microscopic analysis, taking advantage that all mutants were made in fully functional GFP-tagged UapA (Fig. 1d). V227A, I230A, and L234A all showed proper PM localization. In contrast, Ser substitution led to either increased endocytic turnover (e.g. V227S), evident by the appearance of cytosolic foci (static vacuoles and motile early endosomes; Martzoukou *et al.* 2018), or total (e.g. I230S) or partial (e.g. L234S) ER retention, evident by the appearance of perinuclear ER rings (Martzoukou *et al.* 2015). In conclusion, aliphatic Ala substitutions in TMS5 were fully tolerated in respect to folding and PM localization, whereas polar Ser substitutions seem to have led to partial misfolding and thus problematic steady-state localization in the PM. This in turn suggested that UapA-dependent growth phenotypes of V227A, I230A, and L234A mutants could be directly assigned to UapA activity.

PM localization for the Ala-substituted UapA mutants was also examined in the presence of substrates (e.g. uric acid). It has been shown that UapA undergoes activity-dependent, substrate-elicited, endocytic turnover, similarly to several transporters in fungi (Gournas *et al.* 2010; Karachaliou *et al.* 2013). Unlike I230A and L234A, which behaved similar to wt UapA, V227A proved highly resistant to substrate-elicited internalization, remaining stably localized in the PM, similar to previously studied loss-of-function mutants (Karachaliou *et al.* 2013). The significance of this finding becomes apparent next. Finally, V227S showed substrate-elicited endocytic turnover, as might have been expected since it was already vulnerable to endocytosis in the absence of substrate.

To further understand the effect of the mutations constructed, we performed direct transport assays using radiolabeled xanthine (Fig. 1e). I230S and L234S substitutions, as might have been expected due to their negative effect on ER-exit of UapA, led to very low transport activities (∼10% and 22%, respectively). Notice that 22% transport rate in L234S justifies growth on xanthine, as the threshold for growth on purines as N sources has empirically been found to be ∼25% of wt UapA transport rate. Mutant V227S showed wt-like transport rate, in line with its capacity to confer growth at 37°C (uptake assays are performed at 37°C for technical reasons; see Krypotou and Diallinas 2014). Mutants I230A and L234A showed increased rate of xanthine transport (2-times of the wt UapA), in line with growth tests, and stable presence in the PM. Surprisingly, V227A, which scored as a loss-of-function mutant in respect to growth on xanthine, showed 40% ‘accumulation’ of radiolabeled xanthine, compared to wt UapA.

To further investigate the effect of TMS5 mutations on UapA transport function activity, and in particular understand the paradox of the V227A substitution, we measured apparent *K*_m_ values for xanthine (Fig. 1e). Ser substitutions in V227 and L234 affected very little the normal *K*_m_ for xanthine (∼10 vs 6 μΜ of wt UapA). Thus, the apparent partial misfolding in these mutants seems to primarily affect subcellular localization, rather the substrate recognition. We did not measure the *K*_m_ for xanthine of I230S due to lack of significant transport activity. Ala substitutions had variable effects on the affinity for xanthine. Mutant I230A had nearly wt-like affinity for xanthine (∼4.5 μM), whereas L234A and V227A showed 9- and 30-times increased affinities (∼0.7 and ∼0.2 μΜ, respectively).

We also determined the *K*_i_ values of I230A and L234A for oxypurinol, which is highly toxic in these mutants. I230A has similar affinity for oxypurinol compared to wild-type UapA (71 vs 103 μΜ), while L234A has ∼6-times reduced affinity for oxypurinol compared to wild-type UapA (678 vs 103 μΜ). These findings (Supplementary Fig. 2) strongly suggest that hypersensitivity to oxypurinol is not due to increased binding affinity, but to increased capacity of steady-state transport of this drug.

In the case of V227A, the measured very high binding affinity for xanthine did not justify the lack of UapA-mediated growth on xanthine. Thus, the simplest explanation of the disagreement of uptake measurements with growth tests is that V227A mutant *binds* tightly, but is unable to *transport*, xanthine. If so, the amount of radiolabeled xanthine detected in uptake assays of V227A should reflect xanthine “trapped” in UapA molecules present at the PM, rather than bona fidae intracellular accumulation of xanthine. To better understand the function of V227A, we also attempted a *counter-flow* experiment where radioactive xanthine-loaded V227A cells were diluted in a buffer containing a molecular excess of nonradioactive xanthine. This experiment might have allowed us to test whether there is rapid decrease of the steady-state level of cell-associated radioactivity due to direct exchange of the radiolabeled with nonradiolabeled xanthine at the binding site of V227A. However, we did not detect any difference with cells expressing wt UapA (Supplementary Fig. 3), which in accordance with the fact that the binding of radiolabeled xanthine is blocked by low levels of nonradioactive xanthine, the inability of growth in medium with xanthine, resistance to oxypurinol and PM stability, further suggested that radiolabeled xanthine, is irreversibly trapped in V227A.

Given that V227A has acquired a 30-times increased binding affinity for xanthine, we also tested whether it shows increased binding affinities for other nucleobases. Uric acid, oxypurinol, and uracil inhibited the binding of radiolabeled xanthine with *K*_i_ values of ∼11, ∼33, and ∼71 μΜ, respectively, while guanine, adenine, and hypoxanthine showed much lower affinities (*K*_i_ of ∼0.5–1.0 mM; Fig. 1f). Overall, V227A confers increased binding affinities for most nucleobases tested, compared to wt UapA. The most noticeable difference with wt UapA, besides the 30-times increase in xanthine binding is that V227A also binds uracil with at least 15-times increased affinity (71 μΜ vs >1 mM; Goudela *et al.* 2005). Specifically in the case of xanthine, dramatically increased binding affinity seems to block transport cycle, which in turn suggests that UapA is trapped in a substrate-occluded conformation. Noticeably also, xanthine apparent trapping in V227A was shown to depend on H^+^ binding, as the use of the CCCP (Carbonyl cyanide m-chlorophenyl hydrazone) proton gradient uncoupler led to dramatic reduction of radiolabeled xanthine associated with cells. Interestingly also, V227 corresponds to V155 in the homologous xanthine transporter of *E. coli*, namely XanQ. It has been shown that replacement of Val155 with Ile (V155I) leads to very low transport activity, replacement with Met (V155M) leads to 5-times decreased kinetic affinity for xanthine, whereas V155C is inactive (Karena *et al.* 2015). Along with the current findings with V227 mutations, these data reinforce the conclusion that this position in TMS5 is mechanistically important in both UapA and XanQ. Overall, our findings show that the character of aliphatic amino acids on the side of TMS5 that faces the sliding-elevator domain is highly critical for transport dynamics in similar transporters.

### TMS12 mutations

**Fig. 2.**
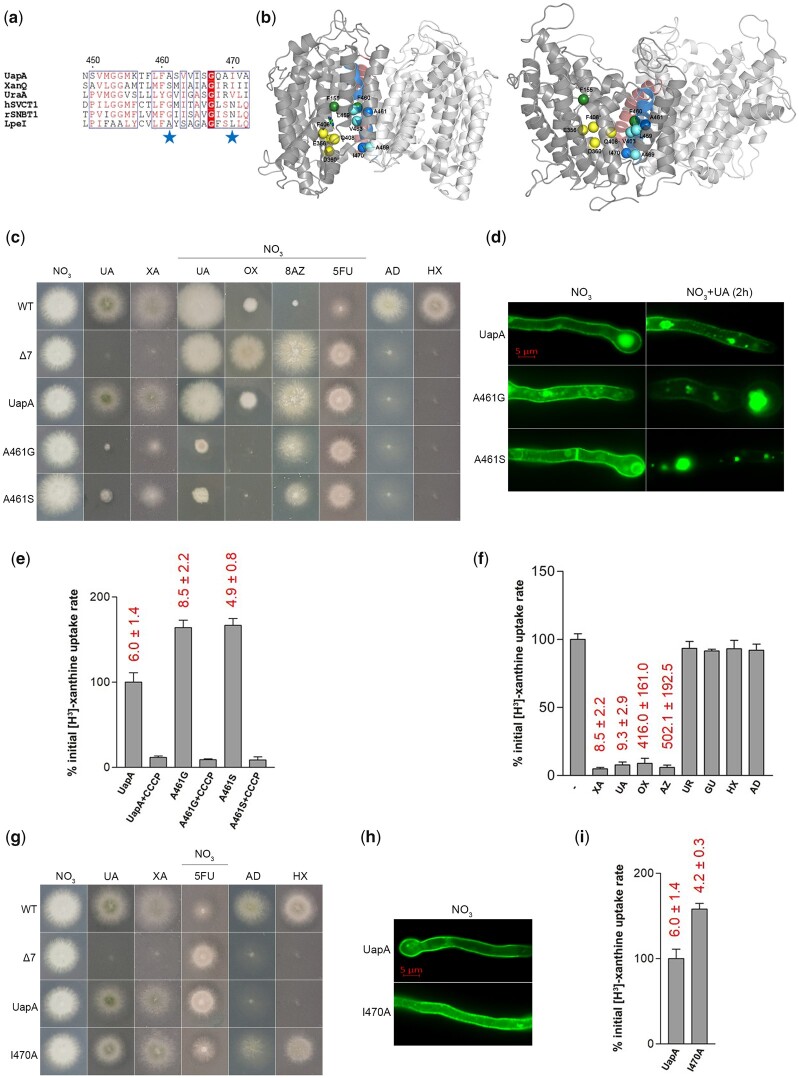
Functional analysis of TMS12 mutants. a) TMS12 sequence alignment of UapA, XanQ, UraA, LPE1, hSVCT1, and rSNBT1. Details on proteins are as in the legend of Fig. 1. b) Topological models of inward-facing (left) and outward-facing (right) conformations of the UapA dimer highlighting TMS12 (blue) and TMS5 (red) at the interface with the core/elevator domain. Blue spheres indicate the positions of the residues mutated (A461 and I470). Yellow spheres represent residues in the core/elevator domain, directly involved in substrate binding (E356 and D360 in TMS8 and F406 and Q408 in TMS10). Green spheres indicate 2 Phe (F150 in TMS3 and F460 in TMS12) crucial for the coordination of the substrate to the respective binding site. Finally, cyan spheres represent residues of TMS12 that are major modifiers of substrate specificity (L459, V463, and A469). For clarity, annotations are shown in one of the 2 UapA monomers (deeper grey). c) Growth tests of UapA mutants A461G, A461S, and control strains on MM. Details are as described in Fig. 1. d) Epifluorescence microscopy showing the subcellular localization of wt UapA and UapA mutants (A461G and A461S) in the presence of ${\text{NO}}_{3}^{-}$ or ${\text{NO}}_{3}^{-}$ + UA (substrate of UapA, 1 mM for 2 h). e) Relative ^3^H-xanthine (0.3 μΜ) transport rates of all mutants analyzed expressed as percentages of initial uptake rates (*V*) compared to the wt UapA (UapA) rate. The time point used for the initial uptake rate is 1 min. Details are as in the legend of Fig. 1. *K*_m_ values (μΜ) for xanthine, estimated as described in Krypotou and Diallinas (2014), are shown at the top of histograms. Results are averages of 3 measurements for each concentration point. SD was less than 20%. f) Relative ^3^H-xanthine (0.3 μΜ) transport rates of strain A461G in the absence or presence of excess (2 mM) unlabeled nucleobases (UR is uracil and GU is guanine), expressed as percentages of initial uptake rates (*V*) compared to the rate of A461G in the absence of unlabeled nucleobases, considered as 100%. *K*_m_ and *K*_i_ values (μΜ) for XA, UA, OX, AZ, and UR are shown at the top of the histograms and were measured as described in Krypotou and Diallinas (2014). Results are averages of 3 measurements for each concentration point. SD was less than 20%. g) Growth tests of UapA mutant I470A and control strains on MM as described in Fig. 1. h) Epifluorescence microscopy showing the subcellular localization of UapA mutant I470A and wt UapA in the presence of ${\text{NO}}_{3}^{-}$ as N source. i) Relative ^3^H-xanthine (0.3 μΜ) transport rates of mutant strain I470A expressed as percentage of initial uptake rate (*V*) compared to the wt UapA (UapA) rate. The time point used for the initial uptake rate is 1 min. UapA initial uptake rate is arbitrarily taken as 100%. *K*_m_ values (μΜ) for xanthine, estimated as described in Krypotou and Diallinas (2014), are shown at the top of histograms. Results are averages of 3 measurements for each concentration point. SD was less than 20%.

TMS12 is a moderately conserved transmembrane segment in UapA homologs (Fig. 2a). Previous unbiased genetic screens and rationally designed mutations have shown that specific replacements of little conserved aliphatic amino acids L459, V463, and A469 facing the interface with the sliding-elevator domain broaden the specificity of UapA toward nucleobase substrates (Kosti *et al.* 2010; Alguel *et al.* 2016). Here, we constructed and functionally analyzed the following TMS12 mutations: A461G, A461S, and I470A. The rationale for mutating A461 was based on a recent mutational analysis of *E. coli* homologs of UapA, and particularly of XanQ, a highly specific xanthine transporter (Tatsaki *et al.* 2021). In XanQ, residue S377, which corresponds to A461 in UapA and faces the TMS12 side toward the dimerization interface (Fig. 2b), proved to be an element critical for specificity as its substitution with Gly changes substrate selectivity enabling non-native substrates to be recognized by XanQ (e.g. guanine). Molecular Dynamic modeling suggests that the S377G replacement tilts TMS12, resulting in a rearrangement of the neighboring F376 relative to F94 (TMS3) and F322 (TMS10) in the substrate-binding pocket. These 3 Phe residues are proposed to pack substrates via pi–pi stacking interactions. Thus, topological modification of this aromatic network might lead to loosening of XanQ specificity. I470 was mutated because it lies at the end of TMS12, at the substrate exit to the cytoplasm (Fig. 2b).

Most interestingly, mutations A461G and A461S led to apparent toxicity of uric acid (Fig. 2c). Toxicity can only be explained by considering uncontrolled accumulation of uric acid, a metabolite known to be cytotoxic at high concentrations.

Also, A461G and A461S conferred hypersensitivity (i.e. forming more compact colonies with reduced diameters and no sporulation) to xanthine, oxypurinol, and 8-azaguanine, the latter being a nucleobase analog not recognized at all by wt UapA. Otherwise, the A461G and A461S remained resistant to the toxic uracil analog 5-FU and were incapable of growing on adenine or hypoxanthine, similarly to the control strain expressing wt UapA (Fig. 2c). Thus, specific substitutions of A461 seem to modify substrate recognition, but mostly affect UapA transport capacity. Noticeably, mutations A461G and A461S conferred increased endocytic turnover of the UapA protein, especially in the presence of native substrates (e.g. uric acid; Fig. 2d). This is often the case with hyperactive mutants that show increased transport rates (Gournas *et al.* 2010).

We confirmed, via direct uptake measurements, that A461 mutations indeed lead to increased, H^+^-dependent, initial transport rate of xanthine, while affecting very little the affinity of UapA for xanthine (Fig. 2e). Noticeably also, A461G led to significantly increased steady-state accumulation of xanthine, reaching a plateau after 1 h of incubation with radiolabeled xanthine, contrasting the 1 min plateau reached via wt UapA activity (Supplementary Fig. 4). We also examined whether A461G affects the binding affinity of uric acid, oxypurinol, 8-azaguanine, adenine, hypoxanthine, guanine, or uracil, by uptake competition assays. Figure 2f shows that the binding profile of A461G is similar, to that of the wt UapA. Thus, uric acid and xanthine are recognized as high-affinity substrates, oxypurinol and 8-azaguanine are recognized by moderate/low affinity, while other purines are not recognized. The main difference with wt UapA is that 8-azaguanine is not recognized at all by wt UapA. Overall, the functional profile of A461G and A461S mutants shows that these mutations do not affect substrate-binding affinities and specificity significantly, but seem to “unlock” UapA transport activity from a control possibly exerted via interactions of TMS12 with residues of the core/elevator domain.

In contrast to A461G and A461S, mutation I470A proved to broaden UapA specificity to include 5-FU, adenine, or hypoxanthine as substrates (Fig. 2g), and did not affect the proper localization and stability of UapA to the PM (Fig. 2h). Uptake assays further showed that I470A has significantly increased transport rate, while conserving nearly wt affinity for xanthine (Fig. 2i). A similar but much more effective enlargement of specificity has previously been obtained with specific mutations in other residues of TMS12 located along the same side of this helix (e.g. mutations in L459, V463, and A469; Kosti *et al.* 2010; Alguel *et al.* 2016). This is very probably due to the distinct topological position of A461 vs L459, V463, and A469, in the α-helix of TMS12. Thus, specific aliphatic residues located along the TMS12 side control both UapA specificity and transport dynamics, with those facing the elevator domain being more prominent modifiers of specificity, while A461 facing the dimerization domain having a more dramatic effect in loosening the transport dynamics.

### Epistatic interactions between TMS5 and TMS12

**Fig. 3.**
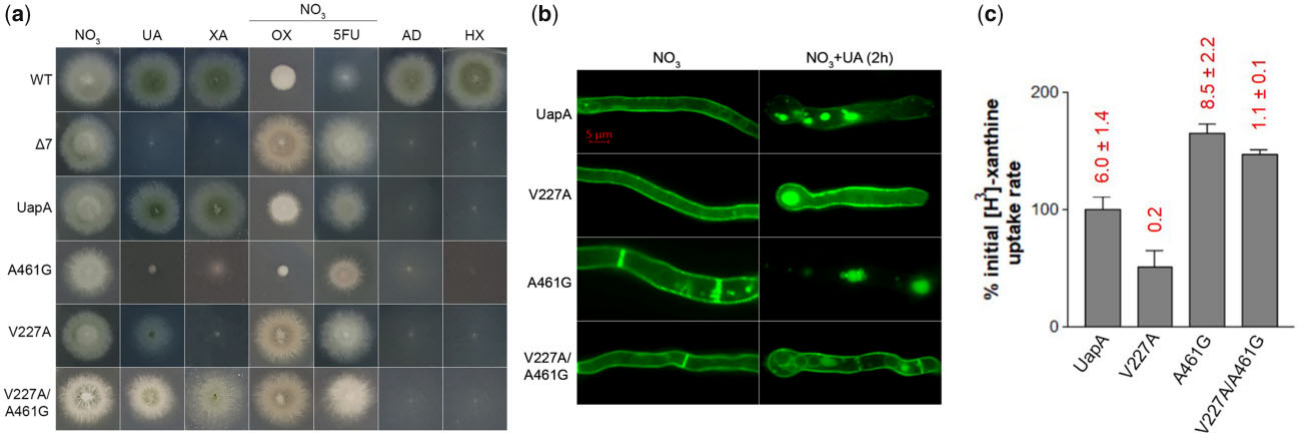
Functional analysis of a strain combining mutations V227A in TMS5 and A461G in TMS12. a) Growth tests of UapA mutants V227A, A461G, V227A/A461G, and control strains on MM as described in Fig. 1. b) Epifluorescence microscopy showing the subcellular localization of UapA mutants V227A, A461G, V227A/A461G, and wt UapA in the presence of ${\text{NO}}_{3}^{-}$ as N source (left column). Two similar experiments with wt UapA, V227A, A461G, and V227A/A461G in the presence of substrate (1 mM UA for 2 h) are shown in the right column. Notice the increased stability of V227A in contrast to the instability/endocytic turnover of A461G. V227A/A461G shows an intermediate phenotype in respect to stability. (c) Relative ^3^H-xanthine (0.3 μΜ) transport rates of all mutants analyzed expressed as percentages of initial uptake rates (*V*) compared to the wt UapA (UapA) rate. The time point used for the initial uptake rate is 1 min. UapA initial uptake rate is arbitrarily taken as 100%. *K*_m_ values (μΜ) for xanthine, estimated as described in Krypotou and Diallinas (2014), are shown at the top of histograms. Results are averages of 3 measurements for each concentration point. SD was less than 20%.

To further understand the role of the 2 most interesting new mutations, V227A and A461G, which seem to have opposing effects on UapA function (i.e. stabilization of a transport-incompetent occluded conformation due to high-affinity substrate trapping vs uncontrolled transport catalysis associated with protein instability/endocytic turnover), we combined these mutations in a single protein. Figure 3 reveals that the functional profile of the double V227A/A461G mutant reveals epistatic relationships of the 2 single mutations, in respect to transport capacity (Fig. 3, a and c), UapA stability (Fig. 3b), and affinity for xanthine (Fig. 3c). Notably, the double V227A/A461G mutant is kinetically distinct from wt UapA and the 2 single mutations, showing positive epistasis in respect to growth on xanthine or uric acid, but not on oxypurinol where the mutant exhibits no signs of sensitivity.

### Conclusion

The present work provides new genetic evidence supporting the critical role of TMS5 and TMS12 helices in the dynamics of transport of a sliding elevator-type transporter. Our results show that modifications of transport dynamics leading to “substrate-lock” (i.e. in TMS5) or “total unlock” (i.e. in TMS12) of sliding of the motile elevator domain, carrying the substrate-binding site, can be achieved via specific conservative mutations of aliphatic amino acids. Genetic changes affecting transport dynamics are also shown to affect transporter stability and specificity. This further suggests that in the course of evolution subtle alterations in TMS5 and TMS12 of elevator transporters might have led to transporters with distinct biophysical properties, transport capacities, or specificity. Noticeably, mutations in conserved aliphatic residues in TMS5 affect mostly the dynamics of transport (negatively or positively), while mutations in partially conserved aliphatic residues in TMS12 lead to uncontrolled transport, which in turn converts UapA into a more promiscuous transporter. Apart from conserved hydrophobic residues forming the interdomain interface, a striking feature concerning helices TMS5 and TMS12 is the presence of a number of highly conserved Gly residues that are invariably conserved in the UapA-related evolutionary cluster, as shown in Karena *et al.* (2015). Importantly, these Gly residues have been shown to be functionally irreplaceable in the XanQ xanthine transporter of *E. coli*, presumably due to crucial need for conformational flexibility at these positions (Karena *et al.* 2015). Finally, the epistatic relationships of TMS5 and TMS12 mutations highlight the difficulty in predicting a priori the overall function of an elevator transporter based solely on the character of amino acid residues directly interacting with substrates. This work also provides a frame for the genetic stabilization of elevator-type transporters for crystallographic or cryo-EM structural analyses.

## Data availability

Strains and plasmids are available upon request. The authors affirm that all data necessary for confirming the conclusions of the article are present within the article, figures, and tables.

Supplemental material is available at *GENETICS* online.

## Supplementary Material

iyac107_Supplementary_Data
