# Supplementary material for: Transmembrane helices 5 and 12 control transport dynamics, substrate affinity, and specificity in the elevator-type UapA transporter
Source: Genetics. 2022 Jul 27;222(1):iyac107. doi: 10.1093/genetics/iyac107 (PMC9434233; doi:10.1093/genetics/iyac107)
Supplement: iyac107_Supplementary_Data [file iyac107_supplementary_data.docx]

**Transmembrane helices 5 and 12 control transport dynamics, substrate affinity and specificity in the elevator-type UapA transporter**

***Dimitris Dimakis^1^, Yiannis Pyrris^1^ and George Diallinas^1,2^****

^1^Department of Biology, National and Kapodistrian University of Athens, Panepistimioupolis, 15784 Athens, Greece.

^2^Institute of Molecular Biology and Biotechnology, Foundation for Research and Technology, 70013 Heraklion, Greece.

*Corresponding author: [diallina@biol.uoa.gr](mailto:diallina@biol.uoa.gr)

**Supplementary Material**

**Figure S1.** Growth tests of UapA mutants V227A and V227S on minimal media (MM) with nitrate (NO_3_^-^) or purines as N source. Nitrate concentration is 10 mM and purine concentration is 0.5 mM. UA is uric acid, XA is xanthine. Tests are performed at 37 °C and pH 6.8. Three control strains are included in the growth tests. WT is a wild-type strain possessing all relative endogenous nucleobase-related transporters. Δ7 is a strain lacking all 7 major nucleobase transporters (Krypotou and Diallinas, 2014). UapA is a Δ7 strain expressing wild-type UapA functionally tagged with GFP. All UapA mutant versions shown are expressed and analysed in the Δ7 strain.

**
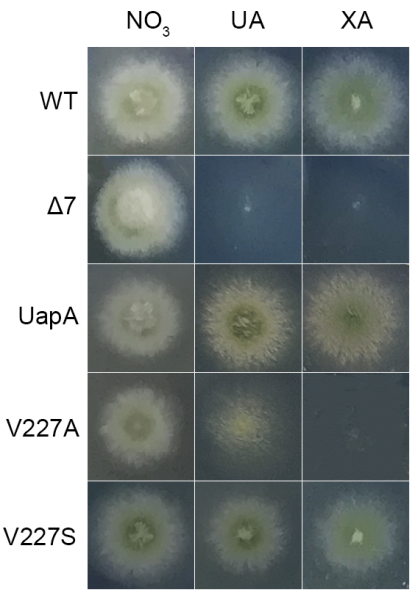
**

**Figure S2.** Relative ^3^H-xanthine (0.3 μΜ) transport rates of strain I230A **(A)** and L234A **(B)** in the absence or presence of excess (2 mM) unlabeled toxic nucleobase analogue oxypurinol (OX), expressed as percentage of initial uptake rate (*V*) compared to the rate in the absence of unlabeled nucleobases, considered as 100%. *K_i_* values (μΜ) for OX are shown at the top of the histograms and were measured as described in Krypotou and Diallinas, 2014. Results are averages of three measurements for each concentration point. SD was less than 20%.


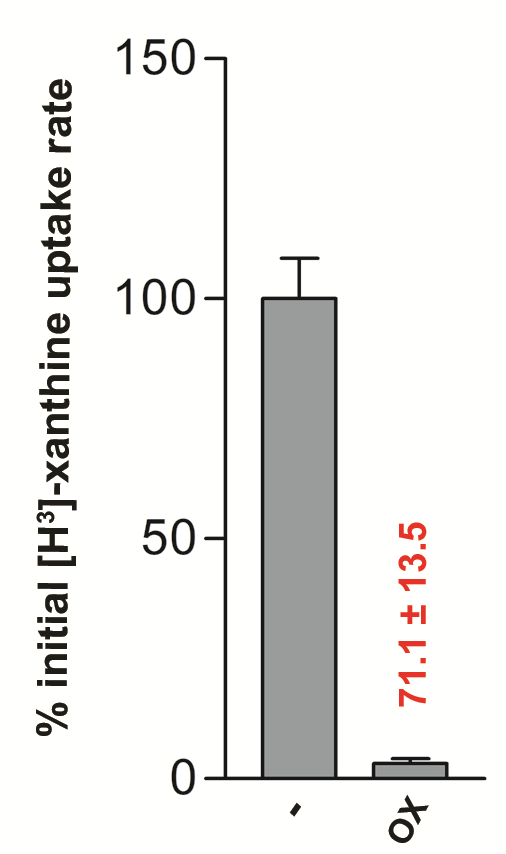

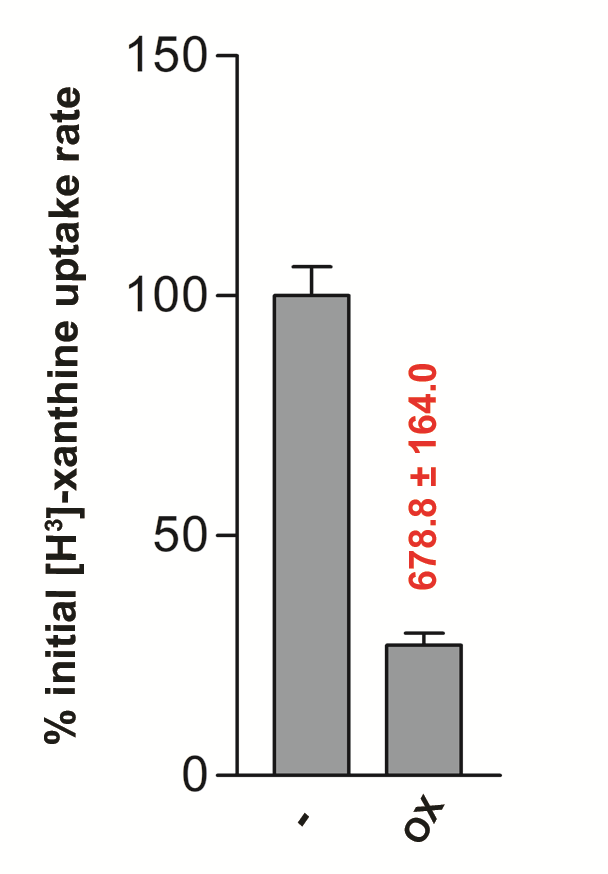


**B**

**A**

**Figure S3.** Counter-flow assay of ^3^H-xanthine loaded cells expressing either wild-type UapA or the V227A mutant. Spores from the strains of interest were initially incubated with radiolabeled xanthine (0.3 μM) for 5 minutes allowing the accumulation of ^3^H-xanthine in cells. The uptake of ^3^H-xanthine was terminated with the addition of 1 mM non-radiolabeled xanthine. Cells were then isolated and washed. The counter-flow assay was performed in the presence of 1 mM non-radiolabeled xanthine for different time periods represented on the X-axis of the histogram. The spores were then isolated, washed and the radioactive counts were measured. The signal of the wild-type UapA strain, which was not incubated with excess non-radiolabeled xanthine (0 min), is considered as 100%. Results are averages of three measurements for each concentration point. SD was less than 20%.

**
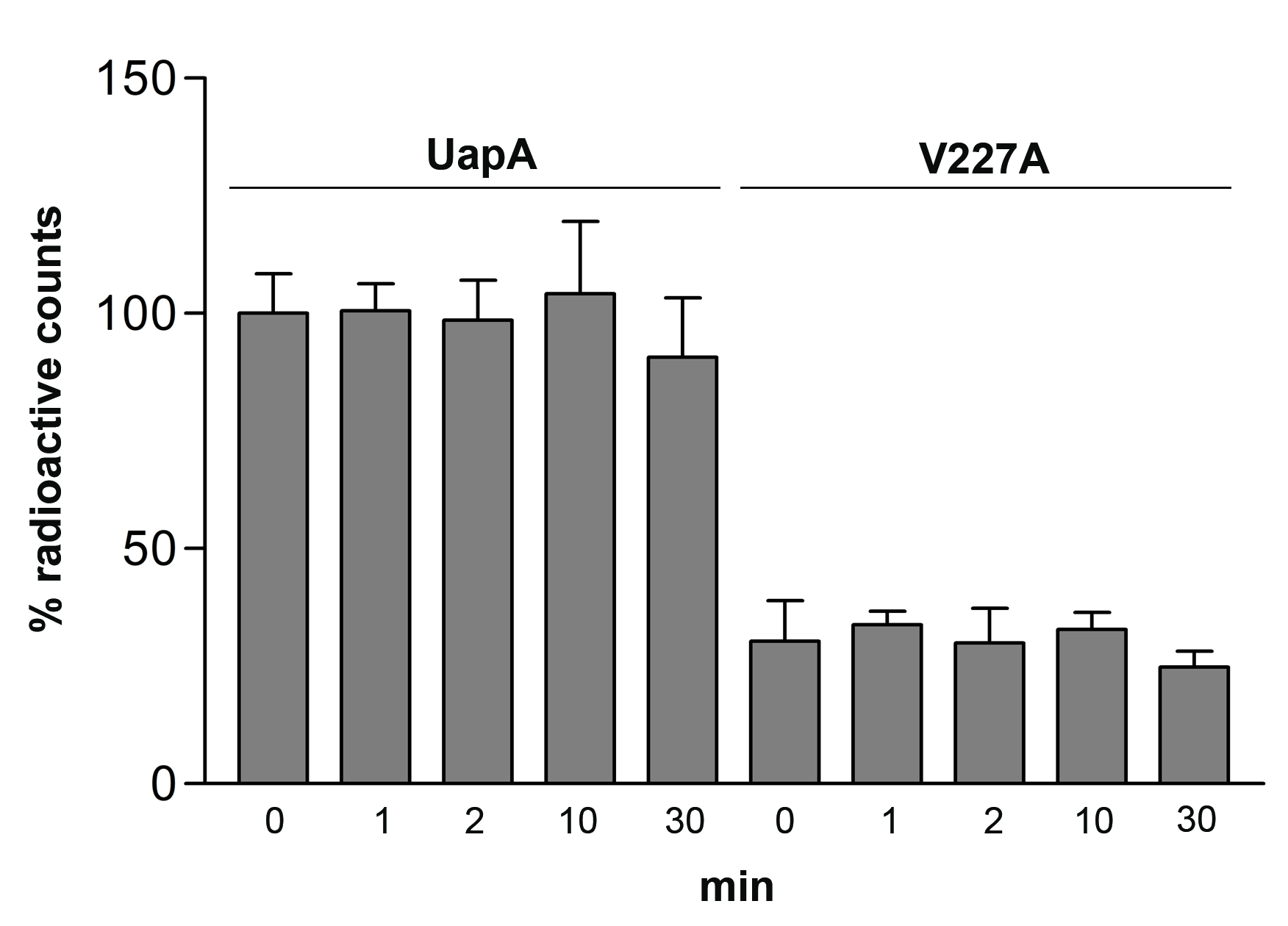
**

**Figure S4.** Relative ^3^H-xanthine (0.3 μΜ) transport accumulation in wt UapA (control) and A461G strains as a time course. Uptake results are averages of three measurements for each concentration point. SD was less than 20%.


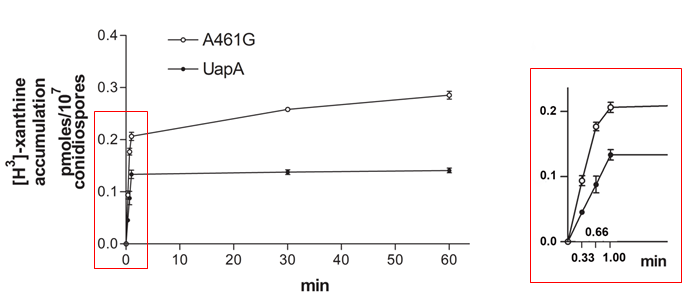


Table S1. Oligonucleotides used in this study. The primers were used for site-directed mutagenesis.

| Mutation | Oligonucleotide sequence of forward primer | Oligonucleotide sequence of reverse primer |
| --- | --- | --- |
| V227A | CATCGTCACTGGTCCCACTGCAATGCTTATCGGGATAAGTCTGATTGG | CCAATCAGACTTATCCCGATAAGCATTGCAGTGGGACCAGTGACGATG |
| V227S | CATCGTCACTGGTCCCACTTCAATGCTTATCGGGATAAGTCTG | CAGACTTATCCCGATAAGCATTGAAGTGGGACCAGTGACGATG |
| I230A | GGTCCCACTGTAATGCTTGCCGGGATAAGTCTGATTGG | CCAATCAGACTTATCCCGGCAAGCATTACAGTGGGACC |
| I230S | CTGGTCCCACTGTAATGCTTAGCGGGATAAGTCTGATTGGAAC | GTTCCAATCAGACTTATCCCGCTAAGCATTACAGTGGGACCAG |
| L234A | GTAATGCTTATCGGGATAAGTGCGATTGGAACTGGGTTCAAAG | CTTTGAACCCAGTTCCAATCGCACTTATCCCGATAAGCATTAC |
| L234S | GTAATGCTTATCGGGATAAGTTCGATTGGAACTGGGTTCAAAG | CTTTGAACCCAGTTCCAATCGAACTTATCCCGATAAGCATTAC |
| A461G | GGGATGAAGACGTTTCTCTTCGGTTCGGTCGTTATTAGCGGACAG | CTGTCCGCTAATAACGACCGAACCGAAGAGAAACGTCTTCATCCC |
| A461S | CGGGATGAAGACGTTTCTCTTCTCTTCGGTCGTTATTAGCGGAC | GTCCGCTAATAACGACCGAAGAGAAGAGAAACGTCTTCATCCCG |
| I470A | CGTTATTAGCGGACAGGCGGCAGTGGCCAAGGCGCCGTTC | TTCCGGCGCCTTGGCCACGGCCGCCTGTCCGCTAATAACG |
